# The screening trap: Why clinical interviews still matter in South African occupational mental health care

**DOI:** 10.4102/sajpsychiatry.v31i0.2461

**Published:** 2025-08-12

**Authors:** Charles H. van Wijk

**Affiliations:** 1 Department of Global Health, Faculty of Medicine and Health Sciences, Stellenbosch University, Tygerberg Campus, Cape Town, South Africa

## Introduction

Common mental disorders (CMDs) are typically clinical diagnoses, made after a comprehensive interview and in-depth history taking. This process, however, demands significant resources, including time and appropriately trained professionals. At the same time, South Africa (SA) grapples with considerable mental health challenges, marked by a high burden of disease and limited resources to manage it.^1^ In such resource-constrained environments, health care providers may increasingly rely on self-report screening questionnaires to guide therapeutic decisions, such as identifying cases of clinical concern, or initiating treatment, or facilitating referrals. This may occur across clinical contexts, whether general medical practice or caring for larger communities often found in occupational healthcare settings.

Despite their utility, reliance on self-report screening scales comes with its own challenges. Respondents more readily amplify symptoms of distress during self-report screening compared to face-to-face clinical interviews.^2,3^ This discrepancy extends to research settings, where estimates of CMD prevalence are typically higher when self-report scales are used,^3^ a finding also documented in SA.^4^ Variability in CMD prevalence within South African samples may thus partly reflect differences in methodologies, such as using screening questionnaires versus interviews.

Self-report scales are understandably appealing in busy general medical settings or in under-resourced occupational and community health care programmes. They enable rapid screening of larger numbers of people, which can make them tempting tools for guiding therapeutic decisions without additional assessment. However, to justify this level of reliance on self-report questionnaires – that is, to use it to inform therapeutic decisions with minimal clinician contact – requires a deeper understanding of their accuracy and correspondence with outcomes derived from assessment by clinical interviews.

Given the well-documented differences between the outcomes of screening tools and clinical assessments, this study examined whether a similar pattern would emerge in the South African occupational health context. The primary aim was to assess the extent to which self-report scales correspond with the outcomes of clinical interviews, thereby evaluating their potential use as stand-alone tools for direct clinical decision-making. A secondary aim was to provide incident prevalence estimates of CMDs in a sample from occupational health surveillance programmes.

## Method

### Sample

This study entailed a retrospective cross-sectional record review of 1800 consecutive cases from occupational mental health surveillance programmes conducted with South African workers in full-time employment during 2023. Participants had a mean age of 35.96 years (± 8.12, range 20–60), with 27.3% being women and 18.2% identifying English as their first language. All participants were skilled workers, and voluntarily completed the surveillance battery in English during their employer-sponsored annual comprehensive health assessments, after providing informed consent.

### Measurements

Participants completed a battery of self-report screening questionnaires and participated in a semi-structured interview with a clinical psychologist who made a diagnosis where appropriate. The clinical diagnoses of major depressive disorder (MDD), generalised anxiety disorder (GAD), post-traumatic stress disorder (PTSD), and alcohol use disorder (AUD) were made according to DSM-5 criteria, while the diagnosis of adjustment disorder (AjD) was made using ICD-11 criteria. The battery is described next.

The Patient Health Questionnaire (PHQ-9) was used to screen for symptoms of depression (Cronbach’s α = 0.84 for this study).^5^ Thresholds for probable diagnosis of MDD have previously been established at ≥ 10 in related local occupational health service samples.^6,7^ The General Anxiety Disorder scale (GAD-7) was used to screen for symptoms of generalised anxiety (Cronbach’s α = 0.87 for this study).^8^ Thresholds for probable diagnosis of GAD have previously been established at ≥ 10 in related local samples.^7^ The Primary Care Post-traumatic Stress Disorder screen for DSM-5 (PC-PTSD-5) was used to screen for the presence of symptoms of PTSD (Cronbach’s α = 0.80 for this study).^9^ Thresholds for probable diagnosis of PTSD have previously been established at ≥ 3 in related local samples, although with poorer positive predictive value (PPV).^7^ The CAGE questionnaire was used to screen for problematic alcohol use (Cronbach’s α = 0.60 for this study).^10^ Thresholds for probable diagnosis of AUD have previously been established at ≥ 2 in related local samples, again with poorer PPV.^7^ The International Adjustment Disorder Questionnaire (IADQ; Cronbach’s α = 0.94 for this study) was used to identify cases of adjustment disorder (AjD), with probable diagnosis of AjD indicated per set criteria from the manual (e.g., presence of psychosocial stressor, and evidence of pre-occupation, failure-to-adapt, and functional impairment).^11^

### Data analysis

All statistical analyses were performed using IBM SPSS for Windows, Version 29. Scale totals and clinical interview outcomes were coded for the presence (or not) of diagnoses across the five conditions assessed. Diagnostic concordance between self-report screening scales and clinical interviews was assessed using Chi-square (χ^2^) analysis. Receiver Operator/operating Curve (ROC) analysis was performed to identify optimal scale cut-off scores, incorporating area under the curve (AUC) and sensitivity and specificity ratios. The study also calculated positive and negative predictive values and incident prevalence estimates.

### Ethical considerations

Ethical clearance to conduct this study was obtained from the Stellenbosch University Health Research Ethics Committee (No. N20/07/078).

## Results and discussion

The results of the χ^2^ analysis, as well as incident prevalence estimates and predictive statistics are reported in Table 1.

**TABLE 1:** Incident prevalence estimates and predictive values for common mental disorders.

Condition	Scale threshold for probable diagnosis			Clinical diagnosis		*χ* ^2^	Predictive values				
	Threshold	*n*	%	*n*	%		AUC	Sensitivity (%)	Specificity (%)	PPV (%)	NPV (%)
Major depressive disorder	PHQ-9 ≥ 10	67	3.7	58	3.2	1090.743*	0.993	84.5	99.0	73.1	99.5
	PHQ-9 ≥ 11	56	3.1	58	3.2	1052.302*	-	75.9	99.3	78.6	99.2
Generalised anxiety disorder	GAD-7 ≥ 10	42	2.3	41	2.3	988.544*	0.987	75.6	99.4	73.8	99.4
	GAD-7 ≥ 11	35	1.9	41	2.3	898.772*	-	65.9	99.5	77.1	99.2
Post-traumatic stress disorder	PC-PTSD-5 ≥ 3	49	2.7	9	0.5	323.228*	0.995	100.0	97.8	18.4	100
	PC-PTSD-5 ≥ 4	22	1.2	9	0.5	575.810*	-	88.9	99.2	36.4	99.9
	PC-PTSD-5 = 5	11	0.6	9	0.5	286.135*	-	44.4	99.6	36.4	99.7
Alcohol use disorder	CAGE ≥ 2	111	6.2	17	0.9	229.427*	0.985	94.1	94.7	14.4	99.9
	CAGE ≥ 3	33	1.8	17	0.9	531.222*	-	76.5	98.9	39.4	99.8
	CAGE = 4	3	0.2	17	0.9	138.744*	-	11.8	99.9	66.7	99.2
Adjustment disorder	IADQ	180	10.0	103	5.7	880.056*	-	95.1	95.2	54.4	99.7

AUC, area under the curve; GAD, generalised anxiety disorder scale; IADQ, international adjust disorder questionnaire; NPV, negative predictive value; PC-PTSD-5, primary care post-traumatic stress disorder screen for DSM-5; PHQ, patient health questionnaire; PPV, positive predictive value.

* , *p* < 0.001.

### Prevalence

The incident prevalence estimates for depression and anxiety were lower than what is typically reported in South African studies.^12,13^ Such studies often reference 12-month or lifetime prevalence rates, which may partially explain the relatively lower figures in this sample. More pertinently, the study specifically focussed on an occupational health context, where employer-provided healthcare included regular screening to identify and stream cases of clinical concern towards appropriate service providers. Such mechanisms enabled early intervention and likely contributed to the lower incidence. In addition, AUD was likely underreported, possibly because of concerns about disclosing alcohol use in occupational health settings.^14,15^

Prevalence data for AjD is sparse, both globally and in South Africa. While relatively common in mental health contexts, data on population prevalence are not known, and it is likely underdiagnosed in the general populace.^16,17^

High co-morbidity was observed in this sample, with 23.7% of cases with positive clinical diagnoses meeting criteria for more than one disorder.

### Self-report screener outcome versus clinical diagnosis

Established thresholds overestimated the incidence of disorders across four of the five scales, namely PHQ-9, PC-PTSD-5, CAGE, and IADQ. Even when adjusted thresholds were used, the χ^2^ analysis still indicated significant differences between scale-determined outcomes and clinical diagnoses (see Table 1). Furthermore, in spite of encouraging AUC figures, poor PPV were reported at previously established thresholds for the PC-PTSD-5, CAGE, and IADQ.

Despite acceptable diagnostic sensitivity and specificity, these self-report scales are primarily designed to identify cases for further assessment. They should not substitute clinical assessment for determining intervention and management. The findings emphasise the need for clinical review and interpretation to inform therapeutic decisions.

### Predictive utility

The PHQ-9 and GAD-7 results confirmed optimal predictive utility – for referral, not diagnosis – at previously established thresholds. The PC-PTSD-5 results suggest optimal predictive utility at a score ≥ 4, at least for this sample. This suggests that different thresholds to inform clinical decision-making may be required across different settings (e.g. primacy care, general practice, occupational medicine, population studies, etc.). Context specific cut-off points could enhance the utility of screening instruments by optimising sensitivity and specificity. In this sample, gender-specific analyses did not result in any meaningful differences in optimal thresholds or other predictive statistics.

The IADQ overestimated cases of AjD, and although it demonstrated good sensitivity and specificity, its PPV was questionable. This again emphasises the necessity of follow-up investigation of self-report outcomes before any clinical decisions can be taken.

### Limitations and future directions

The study’s sample is not representative of the general population, given its focus on an occupational health context. Consequently, findings may not generalise to broader community or primary care settings. In addition, reliance on retrospective data limits the scope for understanding longitudinal trends.

Future research could explore context-specific thresholds and predictive utilities to enhance the accuracy and applicability of self-report scales. Ultimately, screening instruments should complement, not substitute, the expertise of clinicians in providing effective mental health care.

## Conclusion

Screening instruments are valuable tools for identifying individuals who may require further mental health assessment and for streaming them towards appropriate services. However, they cannot substitute clinical judgement and comprehensive evaluation. Over-reliance on these tools for therapeutic decision-making carries the risk of misdiagnosis and inappropriate treatment. Proper assessment remains essential to effective mental health care in the South African context – clinical interviews still matter.
